# Association between physical activity and cognition in Mexican and Korean older adults

**DOI:** 10.1016/j.archger.2020.104047

**Published:** 2020-04-06

**Authors:** Vera Aarsland, Miguel Germán Borda, Dag Aarsland, Elkin Garcia-Cifuentes, Sigmund Alfred Anderssen, Diego Alejandro Tovar-Rios, Camilo Gomez-Arteaga, Mario Ulises Perez-Zepeda

**Affiliations:** a Centre for Age-Related Medicine (SESAM), Stavanger University Hospital, Stavanger, Norway; b School of Medicine, Semmelweis University, Budapest, Hungary; c Semillero De Neurociencias y Envejecimiento, Aging Institute, Medical School Pontificia Universidad Javeriana, Bogotμ, Colombia; d Faculty of Health Sciences, University of Stavanger, Stavanger, Norway; e Department of Old Age Psychiatry, Institute of Psychiatry, Psychology and Neuroscience, King’s College London, London, UK; f Grupo De Neurociencias De Antioquia, Medical School Universidad de Antioquia, Medellin, Colombia; g Department of Sports Medicine, Norwegian School of Sport Sciences, Oslo, Norway; h Unidad Geriatría Hospital Universitario San Ignacio, Bogotá, Colombia; i Universidad Del Valle, School of Statistics, Faculty of engineering Santiago De Cali Valle Del Cauca, Colombia; j Geriatric Medicine Research, Dalhousie University and Nova Scotia Health Authority, Halifax, NS, Canada; k Geriatric Epidemiology Research Department, Instituto Nacional De Geriatria (INGER), Mexico City, Mexico; l Universidad Autónoma de Occidente, Faculty of Basic Sciences, Department of mathematics and statistics, Santiago de Cali Colombia

**Keywords:** Physical activity, Cognition, Aged, Dementia, Cognitive impairment

## Abstract

**Introduction:**

As the world’s population ages, the prevalence of cognitive impairment associated with age increases. This increase is particularly pronounced in Asia and South-America. The objective of this study was to investigate separately the longitudinal association of physical activity and cognitive function in; older adults in Mexico and South Korea.

**Materials and Methods:**

This is a secondary analysis of two surveys, The Mexican Health and aging Study (MHAS) (n = 5853) and Korean Longitudinal Study of aging (KLoSA) (n = 5188), designed to study the aging process of older adults living in Mexico and South Korea. Participants older than 50 years were selected from rural and urban areas achieving a representative sample. Physical activity was assessed using self-report. Cognition was assessed using Cross-Cultural Cognitive Examination (CCCE) and Minimental state examination (MMSE) in Mexico and South Korea respectively. Here we investigate the longitudinal association between physical activity and cognition during 3 years for MHAS and 4 years for KLoSA using multiple linear regression analyses.

**Results:**

The prevalence of physical activity was 40.68 % in MHAS and 35.57 % in KLoSA. In the adjusted longitudinal multivariate analysis, an independent association was found between physical activity and MMSE score OR 0.0866 (CI 0.0266-0.1467 p-value 0.0047) in the Korean older adults, while there was no significant association in MHAS.

**Conclusions:**

Physical activity could have a protective effect on the cognitive decline associated with aging in the Korean population.

## 1. Introduction

As the world population ages, the prevalence of age-associated cognitive decline and dementia increases (Orgeta, Mukadam, Sommerlad, & Livingston, 2019). This is not only detrimental for the affected individuals but also bears a major emotional, physical and economic burden on the health care providers and society. Up to 47 million people worldwide live with dementia today which is expected to double by 2050 (ADI, 2019), and the increase is particularly pronounced in Asia and South-America (UN, 2019). Since therapeutic options for dementia are scarce, measures that prevent or delay this condition is of significant importance.

The rate of cognitive decline associated with aging is highly variable, likely explained by multiple factors, such as the molecular background of the underlying changes in the brain, lifestyle and environmental factors.

Physical activity (PA) is known to improve cardiovascular functions with protective effects on the brain (Erickson et al., 2019; Jia, Liang, Xu, & Wang, 2019; Mandolesi et al., 2018). Also, skeletal muscles can release myokines that induce angiogenesis and promote neuroplasticity, especially in the hippocampus and medial temporal lobe (Iizuka, Machida, & Hirafuji, 2014).

Accordingly, it is not surprising that PA is one of many lifestyle factors identified as a modifiable risk factor for dementia (Livingston et al., 2017), and has been linked to cognitive decline in a dose-response relationship (Sanders, Hortobágyi, la Bastide-van Gemert, van der Zee, & van Heuvelen, 2019). Long term PA has been shown to slow down the progression in Alzheimer disease (Cui, Lin, Sheng, Zhang, & Cui, 2018).

Importantly, most studies of the association between PA and dementia have been conducted in Western countries, whereas few studies are reported in Asia and even less in America Latina. These populations have different cultures, lifestyle habits including dietary and PA habits, and genetic makeup than those previously studied, and thus the association between PA and dementia may differ in these cohorts.

Therefore, we aimed to investigate the longitudinal association of PA and cognitive function in older adults in Mexico and South Korea.

## 2. Methods

### 2.1. Participants

This is a secondary analysis of two national studies; The Mexican Health and Aging Study (MHAS) (Wong, Michaels-Obregon, & Palloni, 2017) and the Korean Longitudinal Study of Aging (KLoSA) (Choi, Son, Cho, Park, & Cho, 2012). The studies were designed to evaluate the aging process in community-dwelling older adults. Both cohorts were representative for the older adults’ population in their countries, the male proportion was 46.5 % in MHAS and 40.23 % in KLoSA, and mean age was 68.6 years (∓ 6.8) in MHAS and 64.4 years (∓ 9.3) in KLoSA.

In both studies, face-to-face interviews were conducted, and subjects were given sets of questionnaires including sociodemographic characteristics, health-related issues, lifestyle habits, and cognitive function. Participants with incomplete data, who refused follow-up or with pre-existing cognitive impairment were excluded.

**Fig. 1.**
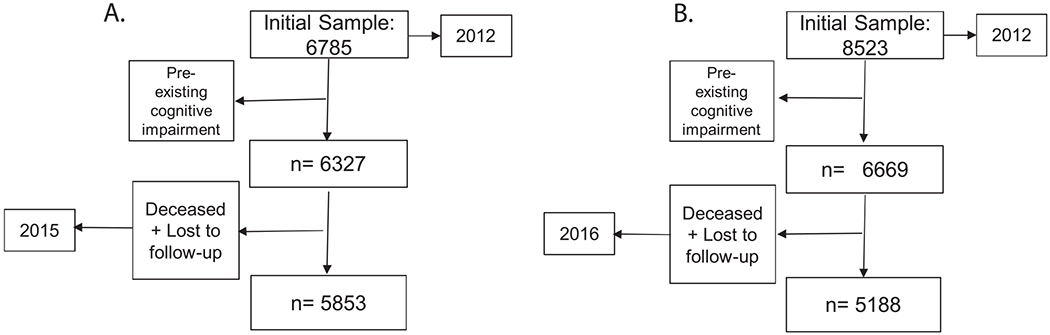
Flowchart of the study sample A. MHAS B. KLoSA.

In the MHAS, participants were selected using a multistage cluster sampling method. In the present study, we used waves 2012 and 2015. Subjects scoring below the cut-off point established through normative data according to years of schooling and age for the Cross-Cultural Cognitive Examination (CCCE) (see below) in 2012 were considered to have pre-existing cognitive impairment and excluded from the analyses Fig. 1.

KLoSA started in 2006, with follow-up every 2-year. A stratified multistage probability sampling was used allowing to obtain a representative sample. The analysis was made on waves between 2012 and 2016. Subjects scoring below 24 on the Mini-Mental State Examination (MMSE) were considered to have cognitive impairment for KLoSA Fig. 1.

The recruitment of participants and methods used in MHAS and KLoSA have been described in detail elsewhere(Borda et al., 2019; Kim, Kim, Kwon, & Park, 2019).

### 2.2. Variables

#### 2.2.1. Physical activity

In MHAS, participants were asked whether or not they took part in vigorous exercise three times a week or more, with vigorous exercise defined as any activity involving physical activity including sports or heavy housework, and were classified as either physically active or physically inactive based on this question. KLoSA participants were asked to report the frequency and duration of PA per week. KLoSA participants were asked to report the frecuency and duration of PA per week, categorized as “physically inactive” if they did < 150 min per week, and defined as ‘physically active’ if they reported more than 150 min. per week. This is a standardized question that has been used extensively in the literature to define self-reported physical activity (Gerst, Michaels-Obregon, & Wong, 2011).

#### 2.2.2. Cognitive functioning

Cognitive function was assessed using cognitive screening tests. In MHAS, CCCE was used, a screening test with > 94 % specificity and > 99 % sensitivity for dementia (Glosser et al., 1993; Mejía-Arango, Wong, & Michaels-Obregón, 2015), including verbal and visual memory, selective attention as well as executive function and motor control. This test is achievable also by illiterates of whom there are many among the older generations in Mexico. The score ranges from 0 to 80, with higher scores indicating better cognitive function. KLoSA subjects were screened using the MMSE a global cognitive measurement of cognitive function with a maximum score of 30 (Folstein, Folstein, & McHugh, 1975). Z-score scaling was used to standardize both scores to have uniformity in the way we display the results.

#### 2.2.3. Confounding variables

Schooling was assessed in years as a continuous variable for MHAS and categorical for KLoSA (no education, less than high school, high school, higher than high school). Health-related variables were collected through self-report. Comorbidities were based on the sum of conditions hypertension, heart disease, respiratory disease, cerebrovascular disease, diabetes, arthritis, and malignancies. Depression was determined according to the MHAS depression questionnaire previously validated for this survey (Aguilar-Navarro, Fuentes-Cantu, Avila-Funes, & Garcia-Mayo, 2007) and in KLoSA with the question: Have you ever had sad, blue or depression feelings that persist longer than 2 weeks during the past one year? Alcohol use was assessed as a self-report question, the answers were re-categorized as a dichotomous variable: current or past drinker and non-drinker.

### 2.3. Statistical analysis

Statistical analysis was completed using R software. Baseline characteristics were reported as frequencies and percentages for categorical variables or mean and standard deviation for continuous variables. For the bivariate analysis, to compare those who were physically active and those who were not, the Chi-square test was applied to categorical variables, whereas t-tests were utilised for continuous variables. Multiple linear regression models were fixed using PA as independent variable and cognition at the follow-up assessement as a dependent variable to allow comparisons between models, and including potential confounders such as age, gender, education, comorbidities, depression, baseline cognitive score and alcohol use as co-variates. The results were expressed as coefficients with 95 % confidence intervals. The primary analysis in both cohorts evaluated whether PA at baseline was associated with the follow-up score on the two cognitive tests. Both cohorts are analysed separately, and due to the different design and methods, we have not attempted to perform any combined analyses.

### 2.4. Ethical issues

The Institutional Review Boards of Ethics Committees of the University of Texas Medical Branch in the United States, the Instituto Nacional de Estadistica y Geografía and the Instituto Nacional de Salud Pública in México approved the MHAS study. The Korean Longitudinal Study of Ageing was approved by the Research Ethics Committee of the Korea Labor Institute. Both surveys data are publicly available, and can be downloaded from the employment survey site with personal information removed. All study participants signed informed consent to participate and to have their data used for research purposes. The study adhered to the ethical guidelines of the Declaration of Helsinki.

## 3. Results

The cohort characteristics are shown in Tables 1 and 2. In Korea, the prevalence of physical activity (those who reported more than 150 min per week) was 35.57 %. Compared to the PA inactive group, the physically active group performed better on MMSE in 2016 (−0.123 ∓ 1.05 vs 0.046 ∓ 0.90, p-value < 0.001). The physically inactive group had a higher proportion of women, less alcohol consumption, fewer years of education and a higher prevalence of depression (Table 1).

In MHAS, the prevalence of those reporting regular PA at least three hours a week was 40.68 %. Compared to the physically inactive group, the physically active group scored higher in CCCE in 2015 (0.099 ∓ 1.01 vs −0.063 ∓ 0.99, p-value < 0.001). They also had more years of education, had less depression and consumed less alcohol (Table 2).

**Fig. 2.**
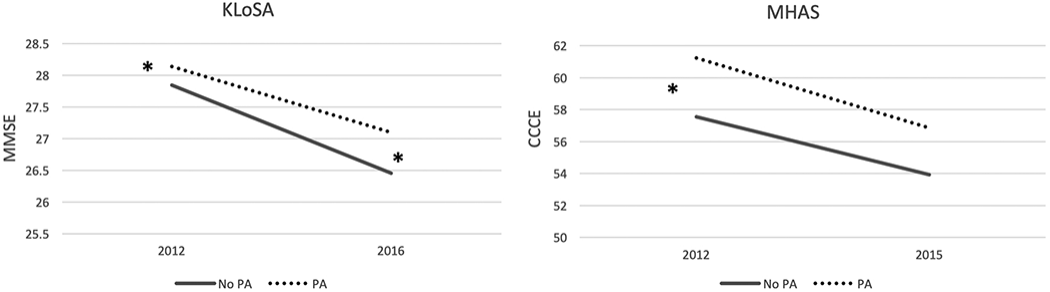
Cognitive test scores in Physical active and physical inactive older adults in the Mexican (MHAS) and South Korean (KLoSA) cohorts. * p-value < 0.05 after adjustments. No PA: No physical active group, PA: Physical active group. MMSE: Mini-mental state examination, CCCE: Cross-Cultural Cognitive Examination.

The baseline and follow-up non-standardised cognitive scores for both populations are shown in Fig. 2.

At the follow-up, an independent association was found in the KLoSA between PA and MMSE score even after adjusting for confounders (0.0661 95 % CI 0.0095; 0.1228, p-value = 0.022) (Table 3).

In contrast, no independent association was found in the MHAS between PA and cognition after adjusting for confounders (0.0119 95 % CI −0.0349; 0.0588, p value = 0.618) (Table 4).

## 4. Discussion

In this study, we investigated the association between physical activity and cognitive function in older adults living in the community in Mexico and South Korea. Our findings suggest a significant association in the Korean population, in which those who were defined as physically active performed significantly better on cognitive testing at follow-up compared to the physically inactive group. In contrast, no significant association between cognition and PA was found in the MHAS cohort. The positive correlation between physical activity and cognition implies that performing regular physical activity, in addition to many other positive health effects, could also have protected against age-associated cognitive decline in Korean older adults.

Little is known about the exact molecular mechanism underlying the relationship between cognitive impairment and physical activity. Previous studies have reported that physical activity and exercise alone may be protective against beta-amyloid related neurodegeneration (Ebrahimi, Majdi, Baghaiee, Hosseini, & Sadigh-Eteghad, 2017).

Some studies have shown that PA stimulates the BDNF, a secretory growth factor that supports neural survival, growth and synaptic plasticity (Hoffmann & Weigert, 2017; Iizuka et al., 2014). Another PA-induced neurotrophin includes PGC-1alpha, which works closely with BDNF: the formation and maintenance of dendritic spines in hippocampal neurons in basal conditions depend on the BDNF-dependent induction of PGC-1alpha after PA (Delezie & Handschin, 2018).

Recent studies describe skeletal muscle as an endocrine organ, releasing myokines that affect the brain. Such molecules proposedly induce angiogenesis linked to plasticity. Among the myokines that have been described to exert their effects on the brain are lactate, IL-6, and irisin. Lactate participates in axonal myelination, angiogenesis and memory formation (Hoffmann & Weigert, 2017). Physical activity also promotes the breakdown of brain glycogen, promoting the lactate-shuttle from astrocytes to neurons, another mechanism implicated in long term memory formation (Delezie & Handschin, 2018).

It draws attention that in the Mexican cohort there was not a significant association between PA and the longitudinal assessed cognition. This can have many possible reasons. Firstly, cognitive measures may be affected by education level, in the MHAS cohort the mean years of education was less than 6 years. In contrast, more than 50 % in KLoSA had at least high school and higher schooling. Secondly, other lifestyle factors, for example diet, may differ and influence the associations between PA and cognition. Oriental diet is rich in nuts, vegetables and fruits, decreasing the risk of cardiovascular and cognitive deterioration (Dong et al., 2016). In contrast, the Mexican diet tends to be unhealthier with higher levels of cholesterol and saturated fat, less vegetables and fruit etc, leading to cerebrovascular risk factors such as high cholesterol, obesity, hypertension, diabetes, which becomes more pronounced with time ans leading to increased risk of cognitive decline (Cruz-Góngora, Cuevas-Nasu, Flores-Aldana, & Shamah-Levy, 2017). A healthier diet is linked with those who are more physically active (Woo, 2000).

Regarding the cognitive tools that were used (MMSE and CCCE), both are screening tools with high sensibility and specificity for dementia, but they are influenced by cultural and educational variables and have limitations for evaluating mild forms of cognitive impairment (Kaltreider & Cullum, 1998). Besides, the results could also be explained by possible less sensitivity to change of the CCCE. However, there is no evidence available that this is true. For Korean population, the method to asses PA was more specific than that for the Mexican population which may generate a tendency of over-or under-reporting PA in Mexican older adults. More sensitive tools for mild cognitive impairment associated with a physical performance examination may increase the feasibility of this correlation.

This study has some limitations. Firstly, this study is based on self-reported measurements of physical activity allowing memory bias. Secondly, variables such as physical activity, cognition, education, and depression were measured using different methods in the studied populations, which may have led to different associations between PA and cognition. Thirdly, PA is incompletely defined regarding the type, duration, or intensity. Although self-reported PA is widely used in the literature, we acknowledge its limitations in obtaining an exact measure of PA.

PA is a complex behaviour that is indeed difficult to assess, and using self-report may limit the validity of the information provided by the participants (recall bias). It is especially challenging to recall light and moderate-intensity physical activity. Thus, self-report may lead to imprecise estimates. Besides, a weak assessment method of PA will dilute the estimated associations because of misclassification of individuals (Andersen, 2004). Hence our results may have underestimated the association between PA and cognition in MHAS. Cognition and Physical activity were assessed in different ways in both surveys. In MHAS PA was defined as “vigorous” based on sports or heavy household, whereas in KLoSA all types of PA were included. However, both high and low exercise intensities have shown the potential to improve cognition and other health domains (Langhammer, Bergland, & Rydwik, 2018; Saez de Asteasu, Martinez-Velilla, Zambom-Ferraresi, Casas-Herrero, & Izquierdo, 2017; Sanders et al., 2019.

Due to several differences between the measurements used in both surveys we did not intend to directly compare the two cohorts and all analyses have been performed separately.

This study encompasses several strengths. It investigates the longitudinal association with a 3-year (Mexico) and 4-year (Korea) follow up, enabling us to see the effects of self-reported physical activity over time. It is also based on cohorts with large and representative cohorts. Lastly, it investigates two different regions in the world both with a growing number of people living with dementia.

Physical activity is a cheap, accessible intervention with a potential to benefit older adults’ health, including cognition. Actions for implementing said intervention and more studies to describe intervention characteristics and precise mechanisms are needed.

## 5. Conclusion

We found a positive longitudinal correlation between physical activity and cognition in South Korean subjects older than 50 years. This implies that performing regular physical activity could have a protective effect on the cognitive decline associated with aging. More studies with objective and standardized measurements in different geographical areas are required to better understand the association between PA and cognition in elderly people.

## Figures and Tables

**Table 1:** Characteristics of PA groups in the KLoSA Study.

Variable	Physically inactive	Physically Active	P-Value	Total
	n (%) or mean ∓ sd	n (%) or mean ∓ sd		n (%) or mean ∓ sd
MMSE score 2015	−0.123 ∓ 1.05	0.046 ∓ 0.90	0.0000	−0.054 ∓ 1.00
Education				
no education	82 (2.43)	16 (0.71)	0.0000	98 (1.47)
< high sch.	1015 (30.02)	443 (19.68)		1458 (21.86)
high sch.	994 (29.40)	841 (37.36)		1835 (27.52)
> high sch.	297 (8.78)	462 (20.52)		1796 (26.93)
Comorbidity	0.87 ∓ 0.98	0.85 ∓ 0.93	0.3056	0.86 ∓ 0.96
0	1499 (44.34)	993 (44.11)	0.3170	3529 (52.92)
1	1103 (32.62)	756 (33.59)		1859 (27.88)
2	538 (15.91)	374 (16.61)		912 (13.68)
3	192 (5.68)	109 (4.84)		301 (4.51)
4	46 (1.36)	18 (0.80)		64 (0.96)
5	2 (0.06)	1 (0.04)		3 (0.04)
6	1 (0.03)	0 (0.00)		1 (0.01)
Alcohol				
Yes	1691 (50.01)	1243 (55.22)	0.0000	2934 (43.99)
No	1690 (49.99)	1008 (44.78)		2698 (40.46)
Depression				
Yes	173 (5.12)	82 (3.64)	0.0090	255 (3.82)
No	3208 (94.88)	2169 (96.36)		5377 (80.63)
Sex				
Male	1524 (45.08)	1159 (51.49)	0.0000	2683 (40.23)
Female	1857 (54.92)	1092 (48.51)		3986 (59.77)
Age	64.62 ∓ 9.62	63.95 ∓ 8.73	0.0067	64.35 ∓ 9.28

Descriptive analysis and bivariate analysis. KloSA. Korean Older adults. Z-score scaling was used for MMSE. MMSE = Mininmental State Examination, PA = Physical Activity.

**Table 2:** Characteristics of PA groups in the MHAS Study.

Variable	Physically inactive	Physically Active	P-Value	Total
	n (%) or mean ∓ sd	n (%) or mean ∓ sd		n (%) or mean ∓ sd
CCCE score 2015	−0.063 ∓ 0.99	0.099 ∓ 1.01	0.0000	0.000 ∓ 1.00
Years of education	5.10 ∓ 4.27	5.63 ∓ 4.61	0.0000	5.30 ∓ 4.41
Comorbidity	1.06 ∓ 0.95	0.85 ∓ 0.88	0.0000	0.98 ∓ 0.93
0	1284 (32.82)	995 (41.20)	0.0000	2279 (36.02)
1	1454 (37.17)	900 (37.27)		2354 (37.21)
2	895 (22.88)	413 (17.10)		1308 (20.67)
3	233 (5.96)	91 (3.77)		324 (5.12)
4	44 (1.12)	15 (0.62)		59 (0.93)
5	2 (0.05)	1 (0.04)		3 (0.05)
Alcohol				
Yes	2429 (93.03)	1364 (89.91)	0.0000	3793 (59.95)
No	182 (6.97)	153 (10.09)		335 (5.29)
Depression				
Yes	1379 (35.25)	762 (31.55)	0.0030	4186 (66.16)
No	2533 (64.75)	1653 (68.45)		2141 (33.84)
Sex				
Male	1540 (39.37)	1402 (58.05)	0.0000	2942 (46.50)
Female	2372 (60.63)	1013 (41.95)		3385 (53.50)
Age	69.20 ∓ 7.05	67.61 ∓ 6.22	0.0000	68.59 ∓ 6.79

Descriptive analysis and bivariate analysis. MHAS. Mexican Older adults. Z-score scaling was used for CCCE. CCCE = Cross-Cultural Cognitive Examination, PA = Physical Activity.

**Table 3:** Cognitive performance of Korean Older adults that were physically active.

Variable	Korea (95 % CI) p-value	
	No adjusted Beta (IC95 %) p-value	Adjusted Beta (IC95 %) p-value
Physical Activity - Yes	**0.1697 (0.1123; 0.2271) 0.000**	**0.0661 (0.0095; 0.1228) 0.022**
Education - No		
< High School		0.8278 (0.5978; 1.0578) 0.001
High School		1.2105 (0.9678; 1.4532) 0.000
> High School		1.0849 (0.8500; 1.3199) 0.000
Comorbility - 0		
1		0.0209 (− 0.0474; 0.0893) 0.800
2		−0.0776 (− 0.1670; 0.0118) 0.283
3		0.0180 (− 0.1252; 0.1612) 0.449
4		−0.0898 (− 0.3909; 0.2112) 0.753
5		−0.5120 (− 2.2283; 1.2043) 0.716
6		1.1360 (− 0.5867; 2.8588) 0.343
Alcohol - Yes		−0.0384 (− 0.1063; 0.0294) 0.170
Depression - Yes		−0.1834 (− 0.3393; - 0.0275) 0.149
Sex - Male		0.0848 (0.0125; 0.1571) 0.019
Age		−0.0298 (− 0.0338; - 0.0258) 0.000
MMSE 2012		0.2534 (0.2241; 0.2828) 0.000

Multivariate analysis for the association between the physically active group and the Longitudinal cognitive performance in Korean Older Adults - KLoSA.

**Table 4:** Cognitive performance of Mexican Older adults that were physically active.

Variable	Mexico (95 % CI) p-value	
	No adjusted Beta (IC 95 %) p-value	Adjusted Beta (IC 95 %) p-value
Physical Activity - Yes	**0.1619 (0.1049; 0.2188) 0.000**	0.0119 (− 0.0349; 0.0588) 0.618
Education		0.0494 (0.0431; 0.0557) 0.000
Comorbidity - 0		
1		−0.0197 (− 0.0728; 0.0332) 0.465
2		−0.0300 (− 0.0922; 0.03211) 0.343
3		−0.0796 (− 0.1875; 0.0281) 0.148
4		−0.1383 (− 0.3697; 0.0930) 0.241
5		−1.5897 (− 2.7350; 0.2450) 0.361
Alcohol - Yes		−0.0654 (−.1474; 0.0166) 0.118
Depression - Yes		−0.0323 (− 0.080; 0.0162) 0.192
Sex - Male		−0.0458 (− 0.0927; 0.0010) 0.055
Age		−0.0276 (− 0.0312; - 0.0239) 0.000
CCCE 2012		0.5418 (0.5116; 0.5719) 0.000

Multivariate analysis for the association between the physically active group and the Longitudinal cognitive performance in Mexican Older Adults – MHAS.
